# Psychological burdens and resilience among Moroccan trainee teachers during the COVID-19 lockdown: A study of challenges and coping strategies

**DOI:** 10.1192/j.eurpsy.2025.1470

**Published:** 2025-08-26

**Authors:** Z. Boumaaize, A. Bouhaba, H. Guider, M. A. Lafraxo, A. EL Alaiki, Y. El Madhi, H. Hami

**Affiliations:** 1 Laboratory of Biology and Health, Faculty of Science, Ibn Tofail University, Kenitra; 2 Higher Institute of Nursing Professions and Health Techniques, Oujda; 3 Regional Center for Education and Training Professions, Rabat, Morocco

## Abstract

**Introduction:**

The onset of the COVID-19 pandemic necessitated the declaration of a global emergency. The pervasive fear of contagion has transformed daily life practices, and lockdown measures globally implemented to mitigate virus transmission have led to a spectrum of adverse psychological effects, including anxiety, sadness, frustration, disorientation, and potential for post-traumatic stress disorder, significantly affecting mental health.

**Objectives:**

This study aims to evaluate the psychological effects of the COVID-19 pandemic on the well-being of trainee teachers.

**Methods:**

A descriptive analysis was conducted on a cohort of 370 Moroccan trainee teachers, with a mean age of 28.30 ± 5.99 years. Data were collected using a self-administered questionnaire designed to assess the presence and extent of psychological distress, along with sociodemographic and professional characteristics, during the lockdown.

**Results:**

The findings revealed that 65.1% of the confined trainees experienced obsessive tendencies, and 40% reported that their daily activities were restricted, affecting their normal life pursuits. Furthermore, 68.3% expressed moderate to high stress levels due to various factors, such as financial and professional concerns, potential loss of family members, or personal hospitalization. Half of the trainees expressed concern for their future prospects and feelings of ennui during these unprecedented times.

**Conclusions:**

The findings reveal the enduring psychological impacts of the COVID-19 pandemic on trainee teachers, with significant stress and psychological disorders noted. This emphasizes the critical need for targeted mental health support and proactive resilience-building within educational systems, not only aiding recovery but also preparing educators for future pandemics.

**Disclosure of Interest:**

None Declared

